# Supplementary material for: Large language models for analyzing open text in global health surveys: why children are not accessing vaccine services in the Democratic Republic of the Congo
Source: Int Health. 2025 Mar 7;17(5):843–52. doi: 10.1093/inthealth/ihaf015 (PMC12406778; doi:10.1093/inthealth/ihaf015)

**Supplementary Information**

***Supp. Figure 1: NLP-approach sensitivity to clustering choice and number of clusters***


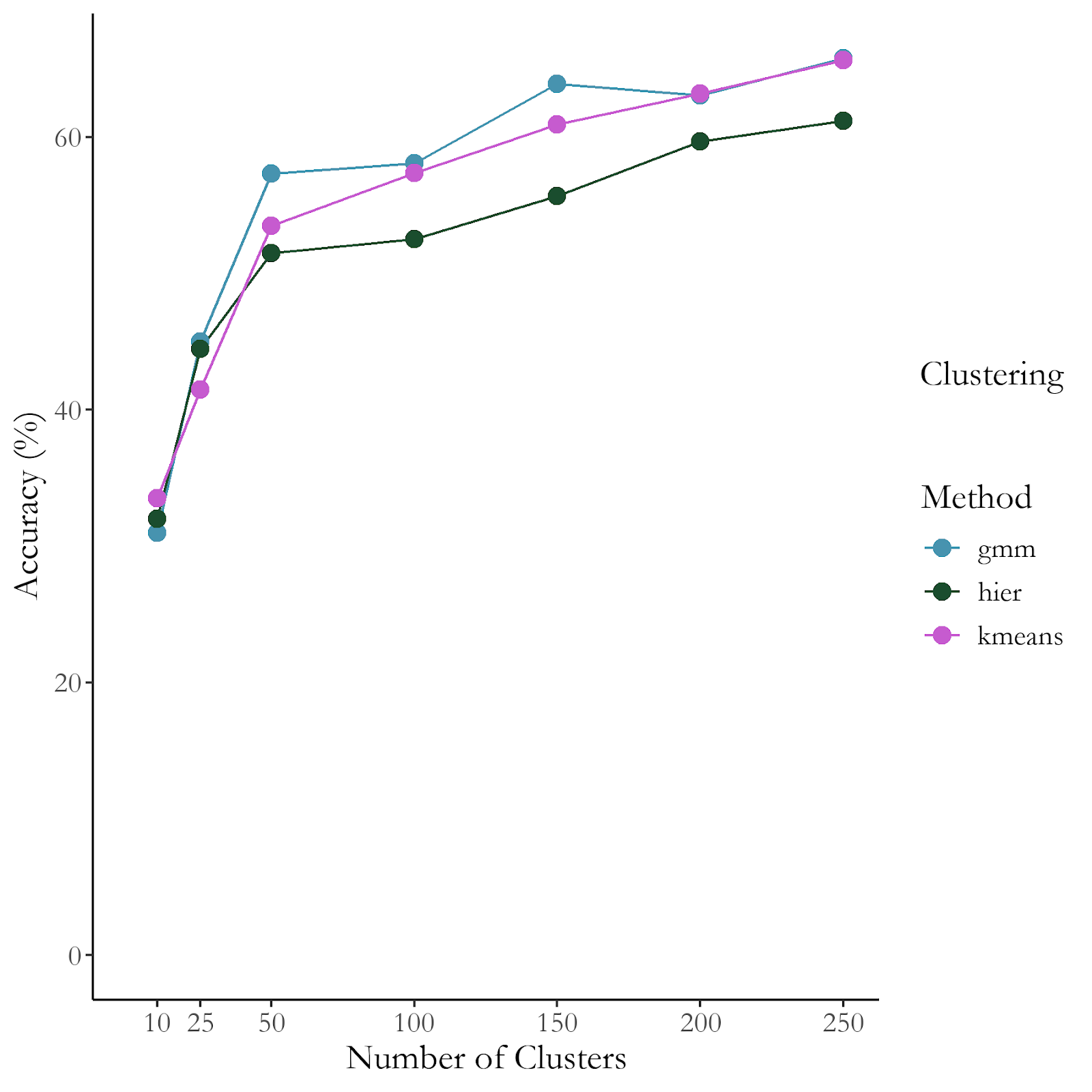

Supplement: ihaf015_Supplemental_File [file ihaf015_supplemental_file.docx]
